# A pilot study evaluating the Calibrated Automated Thrombogram assay and application of plasma-thromboelastography for detection of hemostatic aberrations in horses with gastrointestinal disease

**DOI:** 10.1186/s12917-021-03058-7

**Published:** 2021-11-08

**Authors:** Marie Louise Honoré, Tina Holberg Pihl, Lise Nikolic Nielsen

**Affiliations:** 1 Department of Veterinary Clinical Sciences, Faculty of Health and Medical Sciences (SUND), University of Copenhagen, Hoejbakkegaard Allé 5a, 2630 Taastrup, Denmark; 2 Section for Internal Medicine, Oncology and Clinical Pathology, Faculty of Health and Medical Sciences (SUND), University of Copenhagen, Dyrlaegevej 16, 1870 Frederiksberg C, Denmark

**Keywords:** Equine, Global hemostatic tests, Hypercoagulation, Tissue factor, Platelet poor plasma

## Abstract

**Background:**

Critically ill horses, such as horses with gastrointestinal (GI) disease, often suffer from hemostatic aberrations. Global hemostatic tests examining the initiation of coagulation, clot strength and fibrinolysis, such as the Calibrated Automated Thrombogram (CAT) and plasma-thromboelastography (TEG) have not been evaluated in horses. This study aimed to evaluate CAT and apply plasma-TEG in horses.

Test performance of CAT was evaluated on equine platelet poor plasma with intra- and inter-assay variability (CV) and a heparin dilution curve. To examine clinical performance of both tests, group comparisons were assessed comparing healthy horses, horses with mild and severe GI disease with both CAT and plasma-TEG.

**Results:**

For CAT, intra- and inter-assay CVs were established for lag-time (1.7, 4.7%), endogenous thrombin potential (1.6, 4.6%), peak (2.6, 3.9%) and time to peak (ttPeak) (1.9, 3.4%). Increasing heparin concentrations led to the expected decrease in thrombin generation. In the group comparison analysis, CAT showed significant higher peak (*p* = 0.04) and ttPeak (*p* = 0.008) in the severe GI disease group compared to horses with mild GI disease and healthy horses, respectively. Plasma-TEG showed an increased angle (*p* = 0.032), maximum amplitude (*p* = 0.017) and shear elastic force (G) (p = 0.017) in the severe GI disease group compared to healthy horses.

**Conclusions:**

CAT performed well in horses. Both CAT and plasma-TEG identified hemostatic aberrations in horses with severe GI disease compared to healthy horses. Further studies including more horses, are needed to fully appreciate the use of CAT and plasma-TEG in this species.

**Supplementary Information:**

The online version contains supplementary material available at 10.1186/s12917-021-03058-7.

## Background

Critically ill horses, such as horses with ischemic or inflammatory gastrointestinal (GI) disease, often suffer from marked hemostatic aberrations [1–5]. The most frequent hemostatic aberration is a hypercoagulable state with a decrease in platelet (PLT) count, an increase in thrombin–antithrombin (TAT) complexes, a prolonged prothrombin (PT) and activated partial thromboplastin (aPTT) time, decreased anti-thrombin (AT) and increased D-dimer [1, 2, 4–6]. When this consumption coagulopathy overwhelms the inhibitory system, the coagulopathy is then said to be uncompensated, and an imbalance arises, that can lead to disseminated intravascular coagulation (DIC) and thrombus formation. This hypercoagulable state can however also progress into a hypocoagulable state in the later stages of the disease process due to the depletion of procoagulant factors [3]. This proves that coagulopathies are dynamic and complex pathophysiologic processes. This might explain why one study looking at TEG and conventional hemostatic markers found horses with severe GI disease (ischemic and inflammatory lesions) to be hypocoagulable [3], whereas another study looking at TEG and conventional hemostatic markers suggested horses with severe GI disease (ischemic and inflammatory lesions) to be hypercoagulable [2]. There has however only been moderate progression in the use of more advanced diagnostic global hemostatic tests in horses [2, 3, 7–14], with more conventional hemostatic markers such as  PLT count, aPTT, PT, AT, fibrinogen, and D-dimer concentration still being the most commonly reported parameters [15, 16].

These hemostatic parameters, however, have some limitations since they are snapshots of different aspects of the hemostatic process. Global hemostatic tests such as thrombin generation time or thromboelastography (TEG) on the other hand are dynamic and cover the entire hemostatic process from initiation to clot development and fibrinolysis [17, 18]. Whole blood-TEG has been evaluated in equine medicine in horses suffering from non-strangulating and non-inflammatory GI disorders such as large colon impactions, inflammatory GI disorders such as enterocolitis, and ischemic GI lesions such as intestinal volvulus [2, 3, 7, 19]. One drawback for this method is that TEG should be analyzed with a fixed storage time of ideally 30 min post-sampling in horses to avoid unnecessary time-related aberrations in the TEG parameters [8]. This shortness in time may defer or reduce the applicability of whole blood-TEG in clinical practice as horses are required to be in a hospital setting for the analysis to be performed. If plasma-based global hemostatic tests could replace the whole blood-TEG, this might improve the applicability of the tests in a clinical setting in equine medicine. TEG analyzed with citrated plasma (plasma-TEG) has been investigated in a couple of species. Plasma-TEG was successfully applied in dogs looking at biological variation [20], while in septic pigs plasma-TEG was shown to display a similar increased hemostatic response as identified with whole blood-TEG [21]. In human medicine, plasma-TEG has been applied in studies examining the TEG device, but also in experimental human endotoxemia [22, 23].  Although plasma-TEG is devoid of the cellular components of hemostasis, the coagulation factors and fibrinogen concentration are still present, and changes in test parameters may still be of interest in patients at risk of hemostatic imbalance.

The plasma based thrombin generation time using the Calibrated Automated Thrombogram (CAT) assay, has previously been investigated in both humans [10, 24, 25] and dogs [13, 26]. CAT permits the direct measurement of thrombin generation in a more physiological setting than conventional clotting assays. In humans, it is considered valuable for the study of hypo- and hypercoagulation [10, 27, 28] and has been recommended for assessing patients with venous thromboembolisms [24], evaluating the prognosis in patients with myocardial infarcts [29], and monitoring anticoagulant therapy [10, 13]. An extensive search through the current literature did not identify the use of the CAT assay in horses.

The aim of the present study was, therefore, to evaluate the performance of the CAT assay in equine citrated platelet poor plasma (PPP) and to apply plasma-TEG as an alternative to whole blood-TEG.

Our hypotheses were that the CAT assay would show a high degree of validity and reliability, that both assays would be applicable in equine PPP, and that hemostatic aberrations would be detectable in horses with GI disease compared to clinically healthy horses.

## Results

Demographic data regarding the included horses in the three groups are displayed in Table 1.

**Table 1:** An overview of the demographic data regarding the horses included in the study in the three different groups (clinically healthy, horses with mild gastrointestinal (GI) disease, and severe GI disease). The included parameters are; number of horses, age in years, bodyweight (BW) in kilograms (kg), sex, breed, and diagnosis. ^a^ Values are displayed as mean (minimum-maximum)

	Clinically healthy	Mild GI disease	Severe GI disease
Number of horses	10	9	15
Age ^a^ (years)	11.2 (4–22.8)	8.4 (5.9–12.9)	12.7 (4.8–24)
BW ^a^ (kg)	559.5 (411–687)	492.9 (311–606)	492.5 (139–700)
Sex	8 mares 2 geldings	5 mares 4 geldings	7 mares 8 geldings
Breed	Standardbreds (*n* = 7) Warm bloods (*n* = 2) Unknown (*n* = 1)	Warm bloods (*n* = 5) Icelandic horses (*n* = 2) Pony (*n* = 1) Unknown (*n* = 1)	Warm bloods (*n* = 7) Frisian (*n* = 1) Icelandic horses (*n* = 2) Pony (*n* = 2) Cold bloods (*n* = 2)
Diagnosis	NA	Large intestinal impactions (*n* = 6) Large intestinal non-strangulating displacements (*n* = 3)	Peritonitis associated to the GI-tract (*n* = 2) Acute colitis (*n* = 4) Strangulated small intestine (*n* = 5) Colon torsion (*n* = 1) Severe gastric ulcers (*n* = 1) Ruptured intestine (*n* = 2)

It was possible to use the CAT assay in the horses. Intra-assay coefficient of variation ranged from 1.6–2.6% and inter-assay coefficient of variability from 3.4–4.7% for lag-time, ETP, peak, and ttPeak, respectively (Table 2).

**Table 2:** Imprecision study of the calibrated automated thrombogram (CAT). Intra- and inter- assay coefficients of variation (CV) in percentages established for lag-time, endogenous thrombin potential (ETP), peak and time to peak (ttPeak) are displayed

	Lag-time	ETP	Peak	ttPeak
Intra- assay CV %	1.7	1.6	2.6	1.9
Inter-assay CV %	4.7	4.6	3.9	3.4

**Fig. 1:**
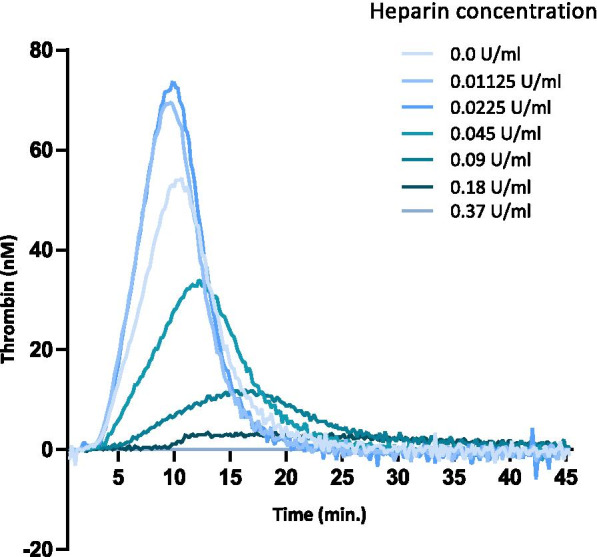
Thrombin generation curves obtained by the calibrated automated thrombogram (CAT) assay for serial dilutions of unfractionated heparin in pooled equine citrated plasma. Thrombin generation decreased with increasing concentrations of unfractionated heparin. Heparin concentrations above 0.18 U/mL completely inhibited thrombin generation and produced a flat-line (0 nM thrombin) and 0.37 U/mL, 0.75 U/mL, 1.5 U/mL and 3 U/mL are therefore not visible on the graph. A heparin concentration of 0.01125 U/ml and 0.0225 U/ml produced a slightly higher peak and endogenous thrombin potential than heparin concentration of 0.0 U/mL

Increasing concentrations of unfractionated heparin resulted in decreasing thrombin generation except for the two concentrations 0.01125 U/ml and 0.0225 U/ml where there was a slight increase in peak and ETP compared to the trace with a heparin concentration of 0.0 U/mL (Fig. 1, supporting information Table 1 s). Heparin concentrations higher than 0.18 U/ml completely inhibited thrombin generation.

Comparing the CAT assay results for the three groups of horses, significant differences were found in peak (*p* = 0.04) and ttPeak (*p* = 0.01) (Table 3).

**Table 3:** Analysis of the calibrated automated thrombogram (CAT) and plasma-thromboelastography (plasma-TEG) between clinically healthy, mild gastrointestinal (GI) and severe GI disease horse groups. Median and range for plasma-TEG and thrombin generation parameters lag-time, time to peak (ttPeak), peak and endogenous thrombin potential (ETP). *P*-value column: one-way ANOVA or a Kruskal-Wallis test depending on normality of data across all three groups with a significance level of < 0.05. ^a^ Post hoc statistical significant difference between healthy and severe GI disease, ^b^ Post hoc statistical significant difference between mild and severe GI disease. SP: split point, R: reaction time, K: clot formation time, α: alpha angle, MA: maximum amplitude, G: shear elastic force, LY30: lysis 30 min, LY60: lysis 60 min (min.)

		Clinically healthy	Mild GI disease	Severe GI disease	***P***-value
**Plasma-TEG**	SP (min.)	11.55 (8.6–16)	11.5 (7.8–16.8)	9.2 (4.2–20.1)	0.53
	R (min.)	13.6 (10.9–20.4)	14.6 (9.3–21.3)	11.3 (4.8–24.5)	0.38
	α (degrees)	18.1 (8.4–25.4)	17.1 (9.5–32.6)	27 (10.2–68.5)	0.016 0.032^a^
	K (min.)	11.85 (9.5–17.9)	10.85 (7.9–19.2)	8.45 (1.4–16.9)	0.14
	MA (mm)	18.45 (11.7–25.2)	22.3 (17–26)	22.1 (13.4–34.4)	0.023 0.017^a^
	G (dynes/cm^2^)	1.1 (0.7–1.7)	1.4 (1–1.8)	1.4 (0.8–2.6)	0.022 0.017^a^
	Ly30 (%)	0 (0–0)	0 (0–0)	0 (0–2.3)	0.53
	Ly 60 (%)	0 (0–0)	0 (0–0)	0 (0–1.1)	0.53
**CAT**	Lag-time (min.)	4.05 (3.4–5.33)	3.72 (2.17–5.67)	3.31 (2.7–7.22)	0.20
	Peak (nM)	32.69 (19.49–47.57)	29.2 (19.63–45.49)	51.46 (15.35–112.4)	0.040 0.04^b^
	ttPeak (min.)	11.87 (10.08–13)	10.28 (8.75–13.89)	9.33 (6.5–13.01)	0.01 0.008^a^
	ETP (nM/min.)	333.1 (270.3–422.1)	292 (179.7–350.8)	346.8 (166.8–468.1)	0.11

**Fig. 2:**
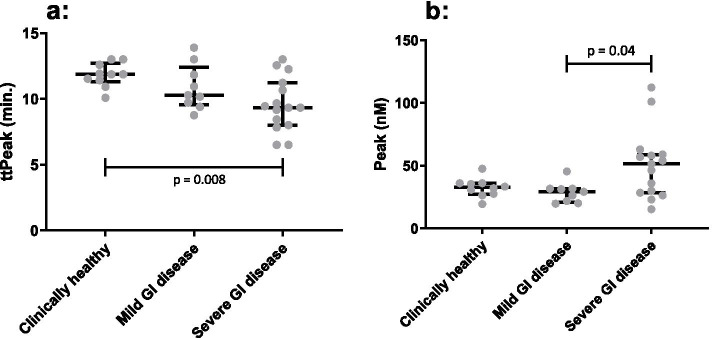
Group comparison between the three groups of horses (clinically healthy, mild and severe gastrointestinal (GI) disease) for the calibrated automated thrombogram (CAT) assay showing a significantly lower time to peak (ttPeak) (*p* = 0.008) in the severe GI group compared to the clinically healthy horses (**a**). Likewise a significantly higher Peak (*p* = 0.04) was found in the severe GI group when compared to the mild GI group (**b**). The horizontal bars are displaying median, 1st, and 3rd quartile

Post hoc analyses identified a significantly higher peak in horses with severe GI disease compared to horses with mild GI disease (*p* = 0.04). A significantly lower ttPeak was seen in horses with severe GI disease compared to the clinically healthy horses (*p* = 0.008) (Fig. 2).

**Fig. 3:**
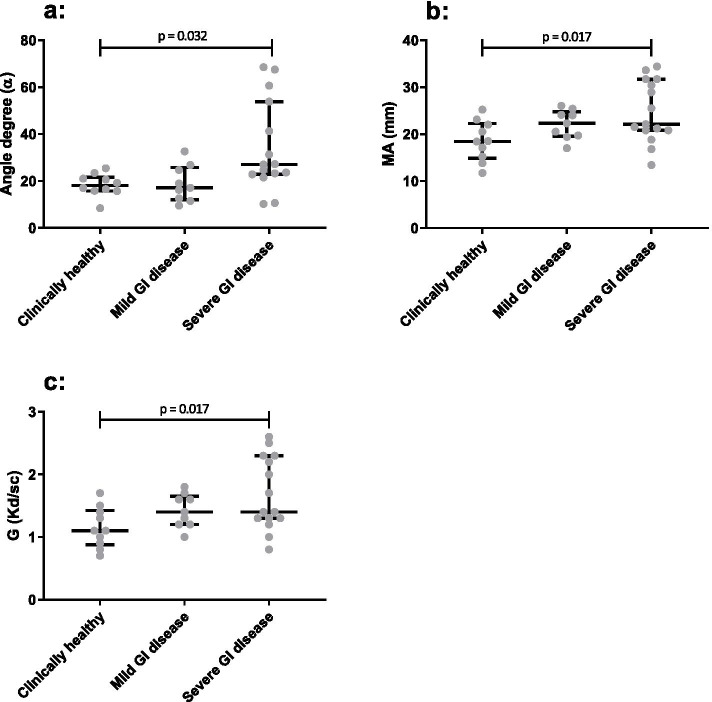
Group comparison between the three groups of horses (clinically healthy, mild and severe gastrointestinal (GI) disease) for plasma-thromboelastography (plasma-TEG) showing a significantly higher angle degree (α) (*p* = 0.032) (**a**), maximum amplitude (MA) (*p* = 0.017) (**b**) and shear elastic modulus strength (G) (*p* = 0.017) (**c**) in the severe GI group compared to the clinically healthy horses. Plots are displaying median, 1st, and 3rd quartile

The plasma-TEG ran successfully in all horses with the exception of not reaching K-values in 12/34 horses. Significant differences were seen for α (*p* = 0.016), MA (*p* = 0.023) and G (*p* = 0.022) between the three groups of horses, with post hoc analysis identifying the differences between horses with severe GI disease and clinically healthy horses (α: *p* = 0.032; MA: *p* = 0.017; G: *p* = 0.017) (Fig. 3 and Table 3).

No significant differences in whole blood-TEG parameters were detected between the three groups of horses (Table 4). When comparing whole blood-TEG and plasma-TEG a significantly lower α (*p* = 0.003), MA (*p* < 0.0001) and G (*p* < 0.0001) were found for plasma-TEG.

**Table 4:** Analysis of whole blood-thromboelastography (WB-TEG) and routine plasma based coagulation tests between clinically healthy, mild gastrointestinal (GI) and severe GI disease horse groups. Median and range for WB-TEG and routine coagulation parameters fibrinogen, activated partial thromboplastin time (aPTT), prothrombin time (PT), anti-thrombin (AT) and d-dimer in healthy horses (healthy control), and horses with mild or severe GI diseases. *P*-value column: one-way ANOVA or a Kruskal-Wallis test depending on normality of data across all three groups with a significant level of < 0.05. ^a^ Post hoc statistical difference between healthy and severe GI disease, ^b^ Post hoc statistical difference between mild and severe GI disease. SP: split point, R: reaction time, K: clot formation time, α: alpha angle, MA: maximum amplitude, G: shear elastic force, LY30: lysis 30 min, LY60: lysis 60 min (min.)

		Clinically healthy	Mild GI disease	Severe GI disease	***P***-value
**WB-TEG**	SP (min.)	12.7 (8.2–17.1)	9.8 (4.4–22.6)	10.8 (5.8–15.9)	0.23
	R (min.)	16.6 (10.5–20.6)	11.6 (5.2–29.5)	13.5 (7.2–19.1)	0.29
	α (degrees)	26.35 (17.2–41.7)	39.3 (13.3–65.4)	35.6 (18.5–54.5)	0.26
	K (min.)	7.15 (4.2–13)	4.9 (2–18.4)	5.4 (3.4–11.8)	0.13
	MA (mm)	51.55 (35–60.1)	56.4 (49.5–73.6)	52.2 (40.9–77.6)	0.18
	G (dynes/cm^2^)	5.3 (2.7–7.5)	7 (4.9–13.9)	5.5 (3.5–17.4)	0.15
	Ly30 (%)	0 (0–1.5)	0.1 (0–0.6)	0 (0–0.9)	0.9
	Ly60 (%)	2.35 (0.2–6.1)	2.9 (0–4.4)	1.3 (0.1–4.4)	0.32
**Conventional haemostatic markers**	Fibrinogen (g/L)	3.17 (2.87–3.53)	3.72 (2.17–5.67)	3.17 (2.57–7.01)	0.14
	aPTT (sec)	47.15 (44.30–57.90)	48.80 (39.30–51.4)	48.00 (40.60–56.30)	0.62
	PT (sec)	13.85 (13.10–14.00)	13.60 (12.70–14.10)	14.10 (12.50–17.30)	0.15
	AT (%)	210.5 (189.00–239.00)	223.00 (195.00–246.00)	197.00 (158.00–260.00)	0.14
	D-dimer	0.08 (0.05–0.1)	0.09 (0.05–0.18)	0.25 (0.1–6.41)	< 0.0001 < 0.0001^a^ 0.0084^b^

There were no differences identified between the 3 groups of horses for fibrinogen concentration, aPTT, PT, or AT. Whereas for D-dimer, a significant difference was found between groups (*p* < 0.0001), with post hoc analysis identifying a difference both between horses with severe and mild GI disease (*p* = 0.0084) and between severe GI disease and clinically healthy horses (*p* < 0.0001) (Table 4).

Correlation analyses between plasma-TEG MA and fibrinogen and between plasma-TEG G and fibrinogen both revealed a moderate and significant Spearman’s correlation coefficient (r) of 0.61 (95% CI: 0.33–0.79, *p* = 0.0001).

## Discussion

CAT ran successfully on equine citrated PPP with satisfactory repeatability based on intra- and inter-assay coefficient of variations for both lag-time, ETP, peak, and ttPeak all below the accepted limit of 5% [30].

In the CAT assay the PPP trigger reagent with 5 pM tissue factor was applied to initiate the thrombin generation process, similarly to what has previously been applied in humans [31] and dogs [32]. The manufacturer provides three different triggering reagents with either 1 pM (PPP low), 5pM (PPP) or 20 pM (PPP high) tissue factor [33]. In human research, the PPP low and high are used in hemophilic patients or patients treated with anticoagulant therapy, respectively. In a recent study in cats [34], all three PPP trigger reagents were tested in depth. Based on performance of the three triggering reagents, PPP low was selected as the most appropriate for the feline species. It is possible that more subtle hemostatic aberrations between healthy horses and horses with severe GI disease could have been detected, if a similar set up had been applied in our study. This needs to be investigated further in future studies.

Endogenous thrombin potential did not differ between horses with severe GI disease and healthy horses. While some consider ETP a more consistently predictive parameter for hemostatic aberrations compared to the remaining CAT parameters, others report an equal importance of lag-time, peak, and ttPeak in the CAT assay [26, 32, 35]. In humans an increased peak and ttPeak has been found as a sign of hypercoagulability in patients with both inherited and acquired hemostatic disorders [36, 37]. We did not expect horses with mild GI disease to have hemostatic aberrations when looking at group comparison, but, surprisingly, it was not possible to distinguish horses with severe and mild GI disease with the global hemostatic tests, apart from peak in the CAT assay. This might have been the result of our two groups with GI disease having disease processes that were too similar concerning their pathophysiology even though we intended to include horses with different severity of inflammation and ischemia. Nevertheless, several parameters were significantly different in both the CAT assay and the plasma-TEG between healthy horses and horses with severe GI disease without being mirrored in the whole blood-TEG. Furthermore, the defrosting and centrifugation process in the CAT assay and plasma-TEG could activate the low number of existing platelets as well as procoagulant platelet micro-particles [38]. Potentially, this could contribute to the initiation phase of plasma-based tests and perhaps affect all parameters in the CAT assay as well as α, K, MA, and G in the plasma-TEG. In people, platelet microparticles are known to contribute to a prothrombotic state in different types of GI diseases [39, 40]. Microparticles were not measured in this study, but it could be speculated that horses with severe GI disease would have a higher concentration of platelet microparticles and thus be more procoagulant than healthy horses. In humans a shorter R and K value and an increased angle degree and MA has been found as a sign of hypercoagulability [41, 42].

Although the plasma-TEG was analyzed in all horses, the K parameter did not produce a reading in 12 of these. This is not a problem of clinical relevance, as it is caused by the fact that the pre-set standard value for K in the TEG machine is defined in the software as the time to clot strength at 20 mm based on human whole blood measurements and due to the narrower tracing of the plasma-TEG, this was not achieved in all cases. In general, the plasma-TEG readings had a lower α, MA, and G compared to whole blood TEG, which likely is due to the different composition of PPP compared to whole blood, probably most importantly the lack of platelets.

Contrary to what have been found in previous studies [2, 3, 43], no differences were identified between the three groups of horses when analyzed with whole blood-TEG. The reason for this is not entirely clear. It is most likely due to the complexity of dealing with coagulopathies [3].

In TEG, MA and G are the comprehensive assessment of the fibrinogen function and concentration combined with the platelet count [44]. When analyzing plasma-TEG using PPP, it could thus be claimed that MA and G of such an assay are no more than an elaborate fibrinogen analysis. In the present study, there was a positive correlation between fibrinogen, MA and G. However, as opposed to plasma-TEG MA and G measured by plasma-TEG, the fibrinogen concentration alone was not able to detect a difference between the three groups of horses. Plasma-TEG has still not been validated extensively in veterinary medicine and whether the plasma-TEG is a more sensitive assay than measuring the fibrinogen concentration on its own remains to be fully elucidated.

The decreased ttPeak and elevated peak for the CAT assay and an elevated angle degree, MA and G for the plasma-TEG in the severe GI disease group corresponds well. Both support a hypercoagulable state which was what we expected looking at the existing literature [2, 6]. For the routine coagulation markers an increased D-dimer was additionally seen in the severe GI disease group, which also supports the finding of a hypercoagulable state [4, 5]. It thus seems that horses with severe GI disease suffers from a hypercoagulable state which in a clinical setting can be evaluated with the use of the CAT assay and plasma-TEG.

The main limitation of the present study is the low number of horses in each group and the variability of the disease processes within each group. The low number of horses was a result of the study being conducted as a pilot study. However, the presented preliminary results do suggest that these global hemostatic tests could be of value in horses and it would be of interest to explore these assays in more detail. Studies could focus on applying the tests to larger groups of horses, horses with visible thromboembolic disease or bleeding disorders to fully examine the potential of both the CAT assay and plasma-TEG in equine medicine.

## Conclusion

The CAT assay performed well in horses with intra- and inter-assay coefficients of variation below the accepted limits and are thus reliable. Additionally the heparin dilution curve showed that the CAT assay is valid in horses. The CAT assay and plasma-TEG were applicable for use in equine citrated PPP and both assays showed hemostatic aberrations in horses with GI disease. Except for ttPeak in the CAT assay, the assays could not distinguish mild from severe GI disease.

## Methods

This study was conducted as a pilot study. All samples were collected at The Large Animal Teaching Hospital at The University of Copenhagen, Denmark, in 2018. Approval was obtained from the ethical board of the Department of Veterinary Clinical Sciences, University of Copenhagen, as was written consent from the owners of the horses.

### Study design

For the evaluation of the CAT assay, intra- and inter-assay coefficients of variation were established in order to establish the reliability of the test in equine PPP. This was performed based on replicate measurements on pooled plasma from 10 clinically healthy horses analyzed 10 times on the same day and once daily for 10 consecutive days. To confirm the capacity of the human CAT assay to measure equine thrombin in PPP and thus establish the validity of the test, unfractionated heparin was added in decreasing concentrations to aliquots of the plasma pool: 0.0, 0.01125, 0.0225, 0.045, 0.09, 0.18, 0.37, 0.75, 1.5 and 3.0 U/mL [45–47].

To examine if these global hemostatic tests deviated in horses with GI disease, group comparisons of both the CAT assay, plasma-TEG, and whole blood-TEG were performed, comparing horses with severe and mild GI disease to healthy horses. The purpose of a group comparison is to detect differences between healthy and clearly sick individuals and is defined as a phase II of test validation [48]. This is performed before proceeding to the more complex phase III involving several different disease and severity groups. We would expect horses with severe GI disease to be hypercoagulable in comparison to clinically healthy horses and horses with mild GI disease.

Whole blood-TEG and the individual hemostatic parameters; D-dimer, fibrinogen, aPTT, PT, and AT were analyzed in the 3 groups of horses in order to compare them to the new global hemostatic tests.

### Horses

Three groups of horses were included in the study: Clinically healthy, mild GI disease, and severe GI disease. The clinically healthy group included adult horses (> 1 year of age) deemed healthy based on clinical examination, complete blood count (CBC) and serum biochemistry profiles, including lactate and the acute phase proteins serum amyloid A (SAA) and fibrinogen concentration being within normal reference intervals. All horses in this group were owned and stabled by The Large Animal Teaching Hospital at The University of Copenhagen. Blood from horses in this group were used in both the intra- and inter-CV studies, the heparin dilution curve and the group comparison. Horses included in the two GI disease groups consisted of adult horses (> 1 year of age) admitted to The Large Animal Teaching Hospital with acute abdominal pain in the period March through June 2018. All samples from hospital patients were collected at admission as part of the initial diagnostic work-up, before placement of intravenous-catheters or administration of any treatments. Horses were assigned to the mild GI disease group if the horses had non-strangulating intestinal obstructions or displacements without signs of secondary inflammation or ischemia of the intestines. Conversely, horses were placed in the severe GI disease group if the horses had inflammatory or strangulating intestinal diseases with ischemia of the intestine. Grouping of the horses was based on clinical signs (heart rate, respiratory rate, temperature and mucous membrane color), CBC, serum biochemistry including SAA, fibrinogen, rectal palpation, naso-gastric intubation, and, where appropriate, abdominocentesis, transabdominal ultrasonography, surgery and post-mortem findings. Horses were considered to have systemic inflammation when there was both clinical or post mortem signs of inflammation like fever or edema in the intestinal wall and inflammatory changes in the blood samples such as leukopenia and increased SAA. Horses were considered to have intestinal ischemia when a segment of the intestine was found to be strangulated and with circulatory changes at surgery or post mortem. Mares included in the study all were reproductively inactive at the sampling point.

### Blood sample handling and routine blood analysis

Blood samples were collected by jugular venipuncture using a 21 g needle and a vacutainer system (BD, Franklin Lakes, NJ). Blood tubes (BD, Franklin Lakes, NJ) were collected in the recommended order [49] starting with the 3.2% 0.109 M sodium citrate tubes in a 1:9 ratio citrate/blood, then serum separator tubes, and finally EDTA tubes. A total of 4 sodium citrate, 1 serum and 1 EDTA tubes were obtained in that order, with the first sodium citrate tube being discarded [49]. The remaining three sodium citrate blood tubes were used for hemostatic assays, while the serum and EDTA tubes were used for the routine biochemistry analysis and CBC (ADVIA 2120i, Siemens Healthcare A/S, Ballerup, Denmark) including a blood smear evaluation, at the hospital’s Diagnostic Laboratorium. The serum and EDTA samples were stored at 4 °C until analysis.

Whole blood TEG with diluted tissue factor (TF) was measured 30 min after sampling in all horses. Platelet poor plasma (PPP) was created within a maximum of 1 h by centrifugation of the sodium citrate tubes at room temperature at 2000 *g* for 15 min [13].. The PPP was stored at -80 °C until batch analysis of plasma-TEG, CAT, d-dimer, fibrinogen, aPTT, PT, and AT. Samples were thawed in a water bath for 4 min at 37 °C and then thoroughly mixed [49]. A platelet count below 10 × 10^9^/L [50] in the PPP was ensured by platelet measurement prior to hemostatic analysis (ADVIA 2120i, Siemens Healthcare A/S, Ballerup, Denmark).

### Hemostatic assays

Thrombin generation was measured with the CAT assay. The following four parameters were evaluated: Lag-time (min), which is the time until 1/6 of the total thrombin concentration is reached; the endogenous thrombin potential (ETP) (nM/min), which represents the total amount of thrombin generated; peak (nM), which is the maximal thrombin concentration, and time to peak (ttPeak) (min) (Thrombinoscope BV, Maastricht, The Netherlands). The Thrombinoscope software is for now only intended for research purposes.

The assay was activated by a manufacturer-supplied trigger solution (PPP reagent) (Triolab AS, Denmark). The measuring interval was set to 20 s, the temperature to 37.5 °C, and the measured time to 45 min. All analyses were performed in triplicates. In each well 80 μl of citrated plasma was added to 20 μl of either the activator solution or thrombin calibrator solution as per the manufacturers recommendation and described previously [13]. The activator solution used contained 5 pM TF and 4 μM phospholipids. Thrombin generation was activated as per the manufacturers (Thrombinoscope BV, Maastricht, The Netherlands) instruction by adding 20 μl of FluCa consisting of Fluo-Buffer containing CaCl_2_ and a fluorescent, and read by an automated highly specific plate reader.

Both plasma-TEG and whole blood-TEG analyses were performed using a computerized thromboelastograph (TEG 5000 Haemostasis Analyzer, Haemoscope Corporation, Illinois, USA). The TEG machines were evaluated prior to daily use with the E-test, which is an electronic quality control. The following TEG parameters were recorded: Split point (SP) which is time to initial conversion of fibrinogen to fibrin; reaction time (R) which is time to initial fibrin clot formation; Clotting time (K) which is the time from initial clot formation until reaching a predetermined level of clot strength (20 mm); Angle (α) which represents the speed of fibrin build up and cross-linking; Maximum amplitude (MA) which represents the maximum clot strength; Shear elastic modulus strength (G) which is a linear function of the MA and considered a measure of global clot strength [51] and degree of fibrinolysis at 30 min (LY30%) and 60 min (LY60%) after MA. The analyses were run as per the manufacturer’s protocol for 2 h. Hypercoagulability for the CAT assay was defined as a shortened lagtime, ttPeak and a higher ETP and Peak compared to the clinically healthy group with one or more altered variable [52]. For TEG hypercoagulability was defined as a shorter R and K value and an increased angle degree, MA, and G (with any ≥1 index being abnormal) compared to the clinically healthy group. For both tests, the opposite situation was defined as hypocoagulability [3, 53–55].

Fibrinogen was measured with a PT-based assay using HemosIL RecombiPlastin on an ACL Top 500 (ILS Danmark, Allerød, Denmark). D-dimer, aPTT, PT, and AT were measured on the automated machine STAGO STA Satellite coagulation analyzer (Triolab, Brøndby, Denmark) with different assays for each parameter according to the manufacturers recommendations. D-dimer was analyzed with STA-Liatest D-Di + which is a photometric antibody-antigen assay based on murine D-dimer antibodies [16, 56], aPTT with STA-Cephascreen using kefalin as an activator which together with PT is a coagulometric assay, PT with STA-NeoPTimal using rabbit thromboplastin, and AT with STA-Stachrom ATIII using bovine thrombin which is a chromogenic assay [16].

### Statistical analysis

Intra- and inter-assay coefficient of variation levels were calculated using Microsoft Excel 2016 (Washington, USA), and levels below 5% were considered acceptable [30]. Remaining analyses were performed using GraphPad Prism 8.3.0 (San Diego, California, USA).

Normality was assessed by the D’Agostino-Pearsons K-squared test. Comparisons between the three groups of horses were performed using a one-way ANOVA or a Kruskal-Wallis test depending on normality of data. As a post hoc test, either Tukey’s multiple comparisons test or Dunn’s multiple comparisons test was applied. Correlation tests (Spearman) between plasma-TEG MA, plasma-TEG G, and fibrinogen concentration were performed in order to evaluate whether these values were connected. The probability of significance was set at a level of < 0.05.

## Supplementary Information

**Additional file 1:** **Table 1s.** Heparin dilution curve performed in the calibrated automated thrombin assay. Lag-time, time to peak (ttPeak), peak and endogenous thrombin potential (ETP) are listed. Note that the thrombin potential is completely inhibited at a heparin concentration of 0.037 U/mL. At the lowest concentration of heparin (0.01125 and 0.0225 U/mL), the lag-time and ttPeak are almost identical to 0.0 U/mL, but with a slight 15–20% increase in both Peak and ETP.

## Data Availability

If deemed relevant or is of interest raw data can be submitted.

## Abbreviations

α: Angle degree (TEG)
aPTT: Activated partial thromboplastin time
AT: Anti-thrombin
BW: Body weight
CAT: Calibrated Automated Thrombogram
CBC: Complete blood count
CV: Coefficients of variation
DIC: Disseminated intravascular coagulation
ETP: Endogenous thrombin potential (CAT)
G: Shear elastic force (TEG)
GI: Gastrointestinal
K: Clotting time (TEG)
LY30%: Fibrinolysis at 30 min after MA (TEG)
LY60%: Fibrinolysis at 60 min after MA (TEG)
MA: Maximum amplitude (TEG)
PLT: Platelet
PPP: Platelet poor plasma
PT: Prothrombin time
R: Reaction time (TEG)
SAA: Serum amyloid A
SP: Split point (TEG)
TAT: Thrombin–antithrombin
TEG: Thromboelastography
TF: Tissue factor
ttPeak: Time to peak (CAT)
WB-TEG: Whole blood-thromboelastography
