# Supplementary material for: A pilot study evaluating the Calibrated Automated Thrombogram assay and application of plasma-thromboelastography for detection of hemostatic aberrations in horses with gastrointestinal disease
Source: BMC Vet Res. 2021 Nov 8;17:346. doi: 10.1186/s12917-021-03058-7 (PMC8573990; doi:10.1186/s12917-021-03058-7)
Supplement: Supplementary file 1 — Additional file 1: Table 1s. Heparin dilution curve performed in the calibrated automated thrombin assay. Lag-time, time to peak (ttPeak), peak and endogenous thrombin potential (ETP) are listed. Note that the thrombin potential is completely inhibited at a heparin concentration of 0.037 U/mL. At the lowest concentration of heparin (0.01125 and 0.0225 U/mL), the lag-time and ttPeak are almost identical to 0.0 U/mL, but with a slight 15–20% increase in both Peak and ETP. [file 12917_2021_3058_MOESM1_ESM.docx]

**TABLE 1s** Heparin dilution curve performed in the calibrated automated thrombin assay. Lag-time, time to peak (ttPeak), peak and endogenous thrombin potential (ETP) are listed. Note that the thrombin potential is completely inhibited at a heparin concentration of 0.037U/mL. At the lowest concentration of heparin (0.01125 and 0.0225 U/mL), the lag-time and ttPeak are almost identical to 0.0U/mL, but with a slight 15-20% increase in both Peak and ETP.

|  | 0.0 U/mL | 0.01125 U/mL | 0.0225 U/mL | 0.045 U/mL | 0.09 U/mL | 0.018 U/mL | 0.037  U/mL | 0.75 U/mL | 1.5 U/mL | 3  U/mL |
| --- | --- | --- | --- | --- | --- | --- | --- | --- | --- | --- |
| Lag-time (min.) | 4.28 | 4.5 | 4.72 | 4.89 | 6.17 | 9.89 | NR | NR | NR | NR |
| ttPeak (min.) | 10.33 | 9.83 | 10 | 12.17 | 16.17 | 16.72 | NR | NR | NR | NR |
| Peak  (nM) | 53.78 | 69.74 | 73.13 | 33.17 | 11.63 | 3.52 | NR | NR | NR | NR |
| ETP (nM*min.) | 431.67 | 482.99 | 508.22 | 326.39 | 180.06 | NR | NR | NR | NR | NR |
